# HIV Protease Inhibitors Act as Competitive Inhibitors of the Cytoplasmic Glucose Binding Site of GLUTs with Differing Affinities for GLUT1 and GLUT4

**DOI:** 10.1371/journal.pone.0025237

**Published:** 2011-09-23

**Authors:** Richard C. Hresko, Paul W. Hruz

**Affiliations:** 1 Department of Pediatrics, Washington University School of Medicine, St. Louis, Missouri, United States of America; 2 Department of Cell Biology and Physiology, Washington University School of Medicine, St. Louis, Missouri, United States of America; Mayo Clinic, United States of America

## Abstract

The clinical use of several first generation HIV protease inhibitors (PIs) is associated with the development of insulin resistance. Indinavir has been shown to act as a potent reversible noncompetitive inhibitor of zero-trans glucose influx via direct interaction with the insulin responsive facilitative glucose transporter GLUT4. Newer drugs within this class have differing effects on insulin sensitivity in treated patients. GLUTs are known to contain two distinct glucose-binding sites that are located on opposite sides of the lipid bilayer. To determine whether interference with the cytoplasmic glucose binding site is responsible for differential effects of PIs on glucose transport, intact intracellular membrane vesicles containing GLUT1 and GLUT4, which have an inverted transporter orientation relative to the plasma membrane, were isolated from 3T3-L1 adipocytes. The binding of biotinylated ATB-BMPA, a membrane impermeable bis-mannose containing photolabel, was determined in the presence of indinavir, ritonavir, atazanavir, tipranavir, and cytochalasin b. Zero-trans 2-deoxyglucose transport was measured in both 3T3-L1 fibroblasts and primary rat adipocytes acutely exposed to these compounds. PI inhibition of glucose transport correlated strongly with the PI inhibition of ATB-BMPA/transporter binding. At therapeutically relevant concentrations, ritonavir was not selective for GLUT4 over GLUT1. Indinavir was found to act as a competitive inhibitor of the cytoplasmic glucose binding site of GLUT4 with a K_I_ of 8.2 µM. These data establish biotinylated ATB-BMPA as an effective probe to quantify accessibility of the endofacial glucose-binding site in GLUTs and reveal that the ability of PIs to block this site differs among drugs within this class. This provides mechanistic insight into the basis for the clinical variation in drug-related metabolic toxicity.

## Introduction

The development and clinical use of HIV protease inhibitors has greatly contributed to the transition of HIV infection from a once fatal disease to its current status as a chronic condition [1]. Tempering enthusiasm for this major advance in HIV treatment is the growing realization that patients treated with combined antiretroviral treatment regimens are at increased risk for the development of pro-atherogenic metabolic side effects including dyslipidemia and insulin resistance [2], [3]. A direct contribution of HIV protease inhibitors to altered glucose homeostasis has been established from several clinical studies [4]. Despite growing awareness of these treatment-related side effects, understanding the mechanisms leading to the development of insulin resistance in treated HIV infection remains incomplete [5]. The ability of PIs to induce insulin resistance in treated patients is not shared by all agents within this drug class. Indinavir and ritonavir appear to have the greatest effect on glucose transport both *in vitro* and *in vivo* whereas newer PIs such atazanavir and tipranavir have minimal to no effect on insulin sensitivity [6], [7]. A direct correlation between the ability of these drugs to block glucose transport *in vivo* and effects on insulin sensitivity in treated patients has been established [8]. Due in part to toxicities and development of viral resistance with existing PIs, the development of safer and more effective antiviral agents remains a high priority. Detailed knowledge of the structural basis of the adverse effects on insulin sensitivity would greatly facilitate these efforts. Greater understanding of the isoform selectivity of these agents would also expand their utility in assessing the contribution of individual transporter isoforms to general glucose homeostasis in both health and disease [9], [10], [11]. Insight into the molecular basis for PI-mediated insulin resistance may also provide a basis for novel approaches to treating the growing worldwide epidemic of type 2 diabetes mellitus.

Previous work has identified the insulin-responsive facilitative glucose transporter GLUT4 as a direct molecular target of several first generation HIV protease inhibitors [12]. While the molecular mechanism by which these drugs acutely and reversibly block GLUT4 intrinsic activity is unknown, the peptidomimetic character found within most PIs has been shown to contribute to this effect [13].

Although the structure of glucose transporters has been inferred by a number of mutagenesis and labeling studies since GLUT1 was first cloned over 25 years ago, to date no crystal structure is available for any of the GLUTs. The proteins are predicted to contain 12 transmembrane spanning alpha helices with both the amino and carboxy termini within the cytoplasm [14]. Extensive kinetic analysis of GLUT1-mediated glucose transport in the erythrocyte membrane has established the presence of two distinct glucose binding sites on either side of the lipid bilayer which cannot be simultaneously occupied [15]. Thus, while zero-trans inhibition experiments have shown that indinavir acts as a non-competitive inhibitor of GLUT4, it remains possible that inhibition is competitive at the cytoplasmic glucose binding site. We have hypothesized that differences in the hydrophobicity of PIs may in part account for differences in the ability of these drugs to inhibit GLUT4 by influencing their ability to access the cytoplasmic surface of the transporter [13].

In order to investigate the ability of PIs to interact with the cytoplasmic surface of GLUT1 and GLUT4, a novel photolabeling-based assay has been developed which allows direct assessment of the influence of drug-protein interactions on the accessibility of the endofacial glucose binding site. In addition to elucidating the mechanism by which PIs inhibit facilitative glucose transport, these data provide a novel means to test for additional protein-protein interactions that may influence glucose homeostasis independent of HIV treatment.

## Materials and Methods

### Materials

2-Deoxyglucose (2-DOG)-1-[^3^H]-glucose was purchased from Sigma (St. Louis, MO). Crixivan (indinavir) was obtained from Merck (White-house City, NJ). Reyataz (atazanavir) was obtained from Bristol-Myers Squibb (Princeton, NJ). Norvir (ritonavir) was obtained from Abbott (Chicago, IL). Tipranavir was obtained from the U.S. National Institutes of Health (NIH) AIDS reference and reagents program. Indinavir and atazanavir were dissolved in water. Ritonavir and tipranavir were dissolved in ethanol. 3T3-L1 fibroblasts were acquired from the American Type Culture Collection. N-[2-[2-[2-[(N-Biotinyl-caproylamino)-ethoxyl]-4-[2-(trifluoromethyl)-3H-diazirin-3-yl]benzoyl]-1,3-bis(mannopyranosyl-4-yloxy)-2-propylamine (PEG-biotincap-ATB-BMPA) was purchased from Toronto Research Chemicals, Inc (Ontario, Canada). GLUT1 polyclonal antibody directed against the carboxyl terminus was a kind gift of Mike Mueckler (Washington University, St. Louis, MO). GLUT4 antibody was custom produced by Invitrogen (Carlsbad, CA) using a peptide corresponding to the 16 carboxy terminal residues of GLUT4.

### 2-Deoxyglucose uptake measurements in 3T3-L1 fibroblasts

3T3-L1 fibroblasts were grown to confluency in Dulbecco's modified Eagle's medium (DMEM) supplemented with 10% calf serum. The uptake of [^3^H]2-Deoxyglucose (50 µM) was measured in Krebs-Ringer phosphate buffer for 6 min at 37°C as previously described [16]. HIV protease inhibitors were added 6 min prior to the start of the reaction. Non-specific uptake measured in the presence of 20 µM cytochalasin B (Sigma) was subtracted from the experimental values.

### 2-Deoxyglucose uptake measurements in primary rat adipocytes

Adipocytes from epididymal fat pads were prepared from male Wistar rats weighing 150 – 200 g (from Charles River Laboratories, Inc., Wilmington, MA) as previously described [17]. Adipocytes were stimulated with 1 µM insulin for 30 min at 37°C prior to use. 2-Deoxyglucose uptakes were performed by adding 80 µl of the cell suspension to 80 µl of Krebs-Ringer bicarbonate Hepes buffer (KRB) (120 mM NaCl, 4 mM KH_2_PO_4_, 1 mM MgSO_4_, 1 mM CaCl_2_, 10 mM NaHCO_3_, 200 nM adenosine, 30 mM Hepes, pH 7.4) containing 3% bovine serum albumin. HIV protease inhibitors / vehicle were added 5 min prior to the initiation of the reaction. Uptakes were initiated with the addition of 20 µl of [^3^H]2-deoxyglucose (final 2-deoxyglucose concentration 50 µM; 0.5 µCi/assay). After 1 min at 37^o^C, reactions were quenched with cytochalasin B to a final concentration of 0.4 mM. The cells were separated by spinning the reaction through dinonylphthalate as previously described [17] and the intracellular radioactivity was quantified by liquid scintillation counting. Non-specific uptake measured in the presence of 20 µM cytochalasin B (Sigma) was subtracted from the experimental values.

### Isolation of low-density microsomes from 3T3-L1 adipocytes

3T3-L1 fibroblasts were grown to confluency and 48 h later subjected to differentiation as previously described [16]. 3T3-L1 adipocytes were used 10 – 14 days after differentiation. Six culture dishes (15 cm) of adipocytes were serum-starved overnight, washed 3 times with ice-cold HES buffer (50 mM Hepes, pH 7.4, 255 mM sucrose, and 1 mM EDTA) containing Roche complete protease inhibitors (Roche Applied Science, Mannheim, Germany), scraped into 5 ml of the same buffer, and then homogenized with either a ball-bearing homogenizer or with a Potter-Elvehjem tissue grinder and a PTFE pestle. The low-density microsome (LDM) fraction was obtained by differential centrifugation as described previously [18]. LDM were resuspended in phosphate buffer (25 mM phosphate, pH 7.4, 100 mM NaCl, 5% glycerol) containing complete Roche protease inhibitors.

### ATB-BMPA photolabeling of LDM

Inhibitors were added to LDM (200 µg protein unless otherwise stated) for 10 min at room temperature. Samples (final volume 110 µl) were then incubated for 10 min at room temperature in the dark with biotinylated ATB-BMPA (50 µM final concentration) and then placed on ice prior to UV irradiation. Reactions were transferred to a 24-well low protein retention culture dish (Costar, Corning, NY) and then irradiated at room temperature 5 cm from a Green Spot UV lamp for 1 min (30 sec of light, followed by 30 sec of darkness, followed by 30 sec of light).

### Isolation and quantification of biotinylated GLUT4 and GLUT1 proteins

20 µl of BSA (1.5 mg/ml final concentration) was added to the UV irradiated samples in a siliconized eppendorph tube. Excess biotinylated ATB-BMPA label was removed using a 0.5 ml Zeba Spin Desalting Column (Pierce). Biotinylated proteins were isolated essentially as described previously [19]. Membranes were solubilized for 30 min at 4°C with 2% Thesit detergent buffer containing protease inhibitors. After centrifugation at 16,000 x g for 10 min, the supernatants were incubated overnight at 4°C with 50 µl of high capacity Streptavidin Agarose resin (Pierce). Precipitates were washed 3 times with 1% Thesit detergent buffer, twice with 0.1% Thesit detergent buffer, and once with phosphate buffer. Biotinylated proteins were eluted at 95 – 100°C for 20 min in 60 µl of 2X Laemmli Sample Buffer containing 40 mM dithiothreitol. Half of the eluted proteins were subjected to SDS-PAGE and transferred to nitrocellulose. Immunoblot analysis was carried out using GLUT4 and GLUT1 specific antibodies and quantified using an Odyssey Infrared Imaging System (LI-COR Biosciences, Lincoln, NE).

### Kinetic analysis of PI effects on ATB-BMPA labeling of LDM

Indinavir (Ind) was added to 50 µg of LDM for 10 min at room temperature. ATB-BMPA (50, 100, 200, 300 µM final concentration) was then added for an additional 10 min (5 min at room temperature, 5 min at 4^o^C). Samples (110 µl final volume) were UV irradiated as described above. After removal of excess label with a desalting spin column, GLUT4 protein was immunoprecipitated from Thesit-solubilized LDM using a GLUT4 C-terminal directed antibody. ATB-BMPA-bound GLUT4 was analyzed by immunoblot analysis using an IR Dye 800 CW fluorescent streptavidin (LI-COR) and quantified with an Odyssey Infrared Imaging System. Identical samples that were not UV irradiated were used to correct for non-specific binding. The free ATB-BMPA concentration was assumed to be equal to the total ATB-BMPA concentration since the label concentration far exceeded the GLUT4 concentration. The data was expressed as Scatchard plots, bound/free versus bound. The B_max_ and K_d_ values were determined from the horizontal intercept and the negative of the slope, respectively. The K_I_ of indinavir was derived from the equation K_d_(apparent)  =  K_d_ (1 + [I]/K_I_) which assumes mutual exclusivity between the two ligands (indinavir and ATB-BMPA) [20].

## Results

### Characterization of ATB-BMPA binding

**Figure 1:**
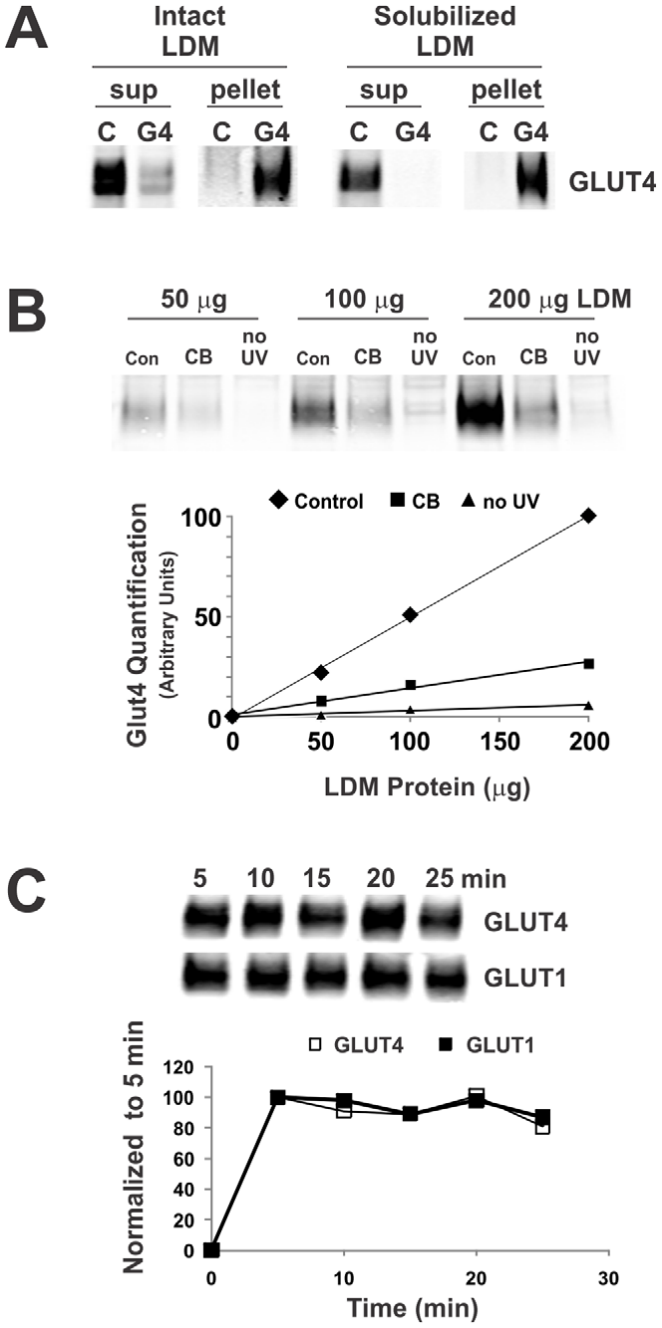
Labeling glucose transporters with biotinylated ATB-BMPA using low-density microsomes. LDM were isolated from fully differentiated 3T3-L1 adipocytes as described in “Materials and Methods”. In (*A*), 175 µg of intact or Thesit-solubilized LDM were immunoprecipitated overnight with 25 µg of control rabbit IgG or 25 µg of GLUT4 C-terminal antibodies precoupled to Protein A-agarose. The supernatant (sup) and pellets were analyzed by immunoblot analysis using a monoclonal GLUT4 antibody (Cell Signaling). In (*B*), ∼1% ethanol (vehicle control) or 20 µM cytochalasin B (CB) were added to the indicated amount of LDM for 10 min at room temperature. Samples were then incubated for an additional 10 min in the dark at room temperature with biotinylated ATB-BMPA (250 µM final concentration). UV irradiation was then performed as described in “Materials and Methods”. Excess photolabel was removed using a spin desalting column. Membranes were solubilized in 2% Thesit detergent buffer and biotinylated proteins were isolated using a high capacity streptavidin agarose resin, and immunoblotted with anti-GLUT4 antibody. GLUT4 protein was quantified using an Odyssey Infrared Imaging System. Membranes containing biotinylated ATB-BMPA that were not irradiated with UV are shown for comparison. In (*C*), biotinylated ATB-BMPA (50 µM final concentration) was added to 70 µg of LDM for varying times prior to UV irradiation. Biotinylated proteins were isolated as described in *A*, and then analyzed by immunoblot analysis using GLUT4 and GLUT1 antibodies. Glut proteins were quantified using an Odyssey Infrared Imaging System.

**Figure 2:**
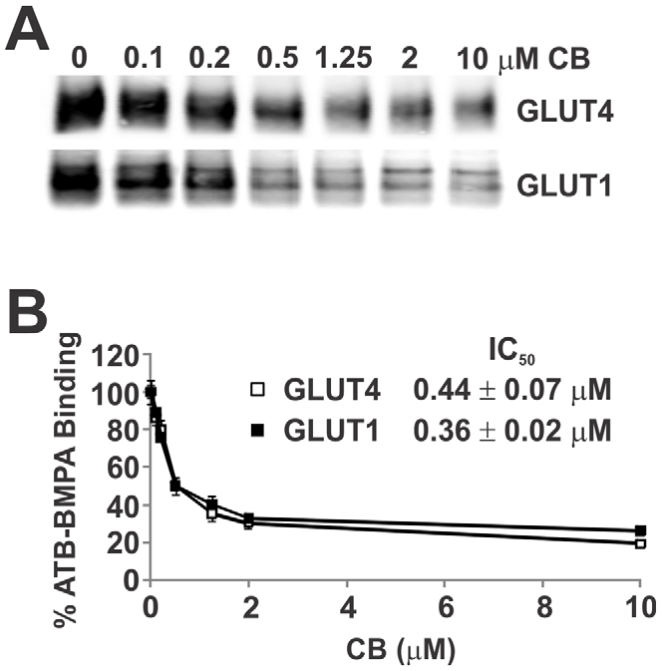
Dose response inhibition of ATB-BMPA photolabeling by cytochalasin B. *A. ATB-BMPA photolabeling.* Ethanol (vehicle control) or cytochalasin B (CB) were added to 200 µg LDM for 10 min at room temperature. Samples were then irradiated with biotinylated ATB-BMPA (50 µM final concentration) as described in “Materials and Methods”. Biotinylated proteins, isolated from detergent solubilized LDM using a high-capacity streptavidin agarose resin, were analyzed by immunoblot analysis using GLUT4 or GLUT1 antibodies. *B. Quantification of ATB-BMPA results.* GLUT proteins were quantified using an Odyssey Infrared Imaging System. Data, normalized to vehicle controls, represent the mean ± S.E. of three independent experiments. Half maximal inhibition (IC_50_) of ATB-BMPA binding to GLUT4 and GLUT1 by CB were determined using a nonlinear least squares analysis (GraphPad Prism, v. 5.0).

The membrane impermeant bis-mannose containing photolabel ATB-BMPA, which inhibits sugar uptake in human erythrocytes and insulin-stimulated rat adipocytes with a K_i_∼350 µM, has been used extensively to quantify cell surface levels of GLUT proteins [21]. ATB-BMPA is generally regarded as an exofacial photolabel and has been used primarily with intact cells [19], [22]. However, if provided accessibility to the endofacial surface of the transporter, ATB-BMPA should theoretically be capable of labeling the cytoplasmic glucose binding site. To test this possibility, ATB-BMPA was used to photolabel low-density microsomes of 3T3-L1 adipocytes. LDM contains small intracellular vesicles of GLUT4 and GLUT1 that translocate and fuse with the plasma membrane (PM) in response to insulin resulting in a dramatic increase in sugar uptake in fat and muscle [23]. In these vesicles, the transporter orientation is inverted relative to that found in the plasma membrane. Specifically, the amino and carboxy termini of GLUT4 and GLUT1 are positioned on the endofacial membrane surface of intact cell but are exofacially oriented in LDM vesicles. Greater than 70% of the GLUT4 vesicles could by immuno-isolated from non-detergent solubilized LDM using a C-terminal directed GLUT4 antibody demonstrating that the vast majority of GLUT4 in LDM was found in this membrane orientation (Fig. 1A). For comparison, using detergent solublized LDM, the efficiency of immunoprecipitation was greater than 95%. As predicted, initial experiments revealed that glucose transporters found in isolated LDM are photolabeled with biotinylated ATB-BMPA in a LDM concentration dependent and cytochalasin B (CB) sensitive manner (Fig. 1B). The time course of incubation of ATB-BMPA with LDM revealed that the photolabeling reaction is at steady state between 5 and 25 min (Fig. 1C). Furthermore, CB reduced ATB-BMPA labeling of LDM in a concentration dependent manner (Fig. 2). The half maximal concentration of inhibition (IC_50_) of CB for ATB-BMPA binding to GLUT4 (0.44±0.07 µM) and GLUT1 (0.35±0.02 µM), is in agreement with previously reported CB inhibition of 2-deoxy-glucose uptake into myotubes (IC_50_ = 0.4 µM) [24].

### Effect of PIs on ATB-BMPA labeling and glucose uptake

**Figure 3:**
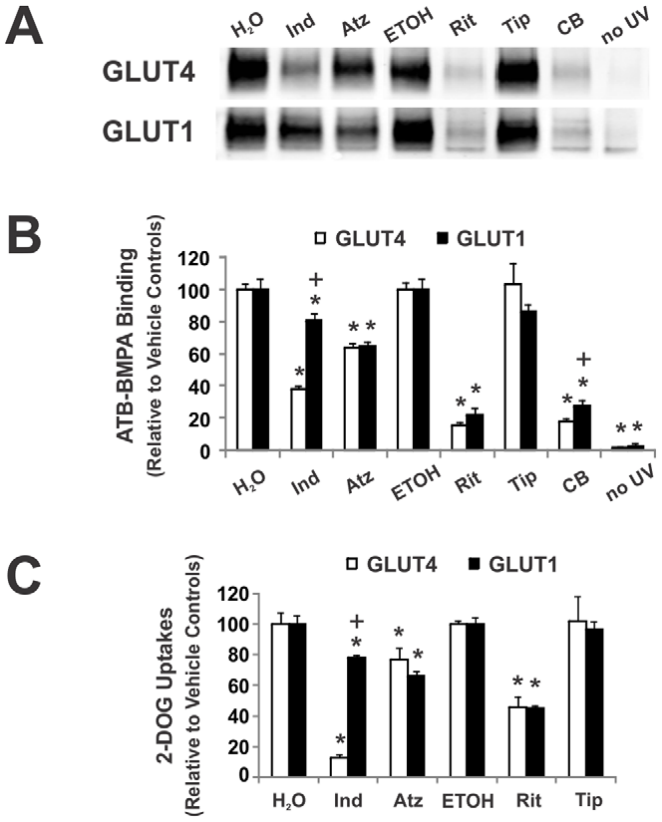
Inhibition of ATB-BMPA photolabeling and glucose uptake by 50 µM HIV protease inhibitors. *A, ATB-BMPA photolabeling*. Indinavir (Ind), atazanavir (Atz), ritonavir (Rit), and tipranavir (Tip) (50 µM final concentrations) were added to 200 µg of LDM for 10 min at room temperature. ATB-BMPA (50 µM final concentration) was then added for 10 min at room temperature prior to UV irradiation. Biotinylated proteins were isolated as described in “Materials and Methods” and then analyzed by immunoblot analysis using GLUT4 and GLUT1 antibodies. The effect of 20 µM cytochalasin B (CB) and no UV irradiation are shown for comparison. *B, Quantification of ATB-BMPA results* GLUT4 and GLUT1 protein were quantified using an Odyssey Infrared Imaging System. Data, normalized to vehicle treated controls, are shown as the mean ± S.E., n = 3; (*), p<0.05 vs. vehicle as determined by the Student's t test; (+), p<0.05 for ATB-BMPA/GLUT4 vs. ATB-BMPA/GLUT1 binding. *C. Glucose Uptakes.* Indinavir (Ind), atazanavir (Atz), ritonavir (Rit), and tipranavir (Tip) (50 µM) were added 5–6 min prior to glucose uptake in insulin-stimulated (1 µM insulin for 20 min) primary rat adipocytes and basal 3T3-L1 fibroblasts. [^3^H]2-Deoxyglucose uptake was measured at 37°C for 1 min in primary rat adipocytes (open bar) and for 6 min in basal 3T3-L1 fibroblasts (filled bar). Data shown as mean uptakes ± S.E. relative to control, n = 4; (*), p<0.05 vs. vehicle; (+), p<0.05 primary adipocytes vs. basal 3T3-L1 fibroblasts.

**Figure 4:**
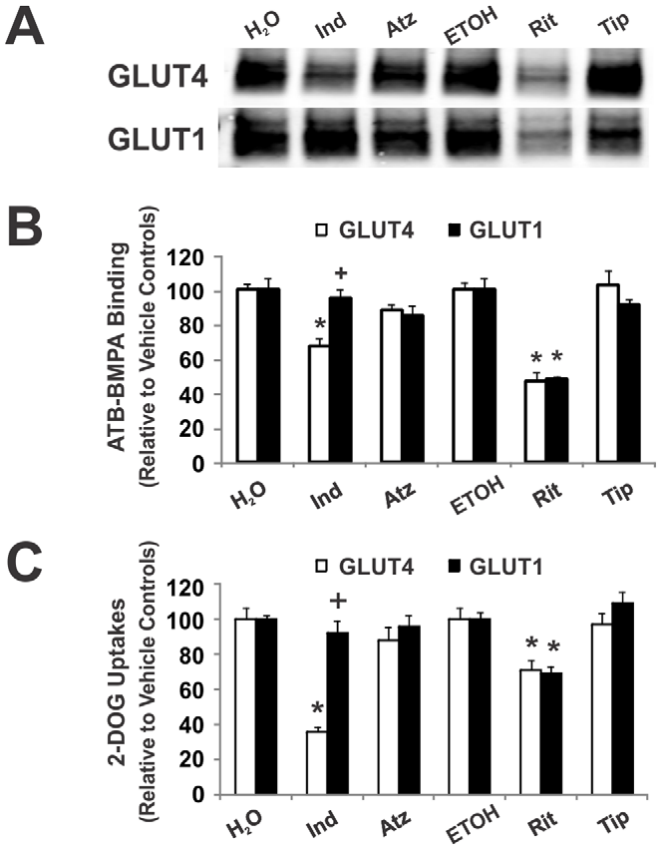
Inhibition of ATB-BMPA photolabeling and glucose uptake by 10 µM HIV protease inhibitors. *A, ATB-BMPA photolabeling*. Indinavir (Ind), atazanavir (Atz), ritonavir (Rit), and tipranavir (Tip) (10 µM final concentrations) were added to 200 µg of LDM for 10 min at room temperature. ATB-BMPA (50 µM final concentration) was then added for 10 min at room temperature prior to UV irradiation. Biotinylated proteins were isolated as described in “Materials and Methods” and then analyzed by immunoblot analysis using GLUT4 and GLUT1 antibodies. *B, Quantification of ATB-BMPA results* GLUT4 and GLUT1 protein were quantified using an Odyssey Infrared Imaging System. Data, normalized to vehicle treated controls, are shown as the mean ± S.E., n = 3; (*), p<0.05 vs. vehicle as determined by the Student's t test; (+), p<0.05 for ATB-BMPA/GLUT4 vs. ATB-BMPA/GLUT1 binding. *C. Glucose Uptakes.* Indinavir (Ind), atazanavir (Atz), ritonavir (Rit), and tipranavir (Tip) (10 µM) were added 5–6 min prior to glucose uptakes in insulin-stimulated (1 µM insulin for 20 min) primary rat adipocytes and basal 3T3-L1 fibroblasts. [^3^H]2-Deoxyglucose uptake was measured at 37°C for 1 min in primary rat adipocytes (open bar) and for 6 min in basal 3T3-L1 fibroblasts (filled bar). Data shown as mean uptakes ± S.E. relative to control, n = 4; (*), p<0.05 vs. vehicle, (+), p<0.05 primary adipocytes vs. basal 3T3-L1 fibroblasts.

Given the tight correlation between the IC_50_ values for photolabel binding to GLUT1 and GLUT4 and glucose transport inhibition by CB, the ability of HIV protease inhibitors (PIs) to similarly alter ATB-BMPA labeling and 2-DG uptake was determined at two different concentrations of PI, 50 µM (Fig. 3) and 10 µM (Fig 4). Photolabeling experiments were carried out with LDM of 3T3-L1 adipocytes to examine the effect of the PIs on ATB-BMPA binding to both GLUT4 and GLUT1. Uptake experiments were conducted in two mammalian cells, 3T3-L1 fibroblasts which are known to express only GLUT1 and in primary rat adipocytes which predominantly express GLUT4 (>90%). Previous results [25] along with our current findings (Fig. 3C and 4C) indicate that indinavir is a selective inhibitor of GLUT4 transporter activity when compared to GLUT1. The selectivity of indinavir for GLUT4 was also confirmed in the ATB-BMPA/LDM photolabeling experiments (Fig. 3B and 4B). Using a therapeutic concentration of indinavir (10 µM), both ATB-BMPA binding and transporter activity were reduced for GLUT4 while GLUT1 was unaffected (Fig. 4). At a higher indinavir dose (50 µM), GLUT1 was also affected but a lesser extent than GLUT4 (Fig. 3).

Unlike indinavir, ritonavir was found to be nonselective for inhibition of both GLUT1 and GLUT4 ATB-BMPA labeling and glucose uptake at both drug doses studied (Fig. 3 and 4). Atazanavir was also a non-specific GLUT inhibitor at the 50 µM dose (Fig. 3). At a therapeutic level, however, atazanavir had no effect on either transporter (Fig. 4). This result is consistent with published reports that therapeutic doses of atazanavir do not inhibit glucose uptake *in vitro* or *in vivo* [26], [27]. Indinavir, ritonavir, and atazanavir all contain a core peptidomimetic structure and all affected GLUT4 and GLUT1 at the 50 µM dosage. Newer PIs like tipranavir have a non-pepidomimetic structure. Interestingly, tipranavir had no effect on the GLUT transporters at either dosage supporting the requirement of peptidomimetic structure for GLUT binding (Fig. 3 and 4).

### Protease inhibitor IC_50_ values for ATB-BMPA labeling of GLUT4 and GLUT1

**Figure 5:**
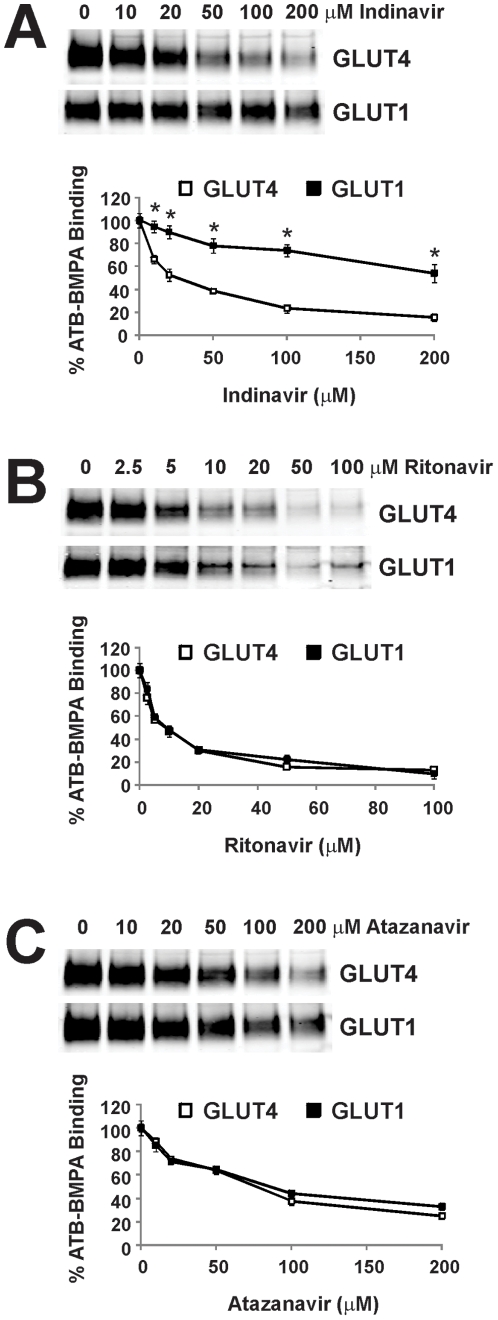
Indinavir but not ritonavir or atazanavir selectively inhibits ATB-BMPA binding to GLUT4 relative to that of GLUT1. Indinavir, ritonavir, and atazanavir were added to 200 µg of LDM for 10 min at room temperature. ATB-BMPA (50 µM final concentration) was then added for 10 min at room temperature prior to UV irradiation. Biotinylated proteins were isolated as described in “Materials and Methods” and then analyzed by immunoblot analysis using GLUT4 and GLUT1 antibodies. GLUT4 and GLUT1 protein were quantified using an Odyssey Infrared Imaging System. Data, normalized to vehicle treated controls, are shown as the mean ± S.E., n = 3; (*), p<0.05 for ATB-BMPA/GLUT4 vs. ATB-BMPA/GLUT1 binding as determined by the Student's t test.

To further characterize the effects of the PIs on ATB-BMPA photolabeling of GLUT4 and GLUT1, PI dose response experiments were conducted with indinavir, ritonavir, and atazanavir. The dose response curves clearly show that indinavir selectively inhibits GLUT4 at all PI concentrations tested while ritonavir and atazanavir were non-selective (Fig. 5). IC_50_ values were determined from the dose response data using a non-linear least squares analysis (Table 1). The GLUT4 and GLUT1 IC_50_ values for indinavir of 20.7 and 178 µM, respectively, is relatively consistent with the IC_50_ values for the inhibition of 2-deoxyglucose uptake by indinavir in primary rat adipocytes (IC_50_ = 11 µM) and 3T3-L1 fibroblasts (IC_50_ = 241 µM). The IC_50_ values for ritonavir were ∼7 µM for both transporters indicating that ritonavir again is a non-selective GLUT inhibitor but also more potent than indinavir. In fact, since ritonavir is poorly soluble in aqueous solutions, the true IC_50_ values for ritonavir may actually be lower. The IC_50_ values for atazanavir were ∼60 µM for both GLUTs indicating both the non-selective nature of the inhibition and that atazanavir inhibits GLUT4 less but GLUT1 more than indinavir.

**Table 1:** Half-maximal inhibition (IC_50_) for ATB-BMPA binding to GLUT4 and GLUT1 by PI's.

	GLUT4	GLUT1
Indinavir	20.7±3.9 µM	178±43 µM*
Ritonavir	7.0±1.1 µM	7.9±0.4 µM
Atazanavir	66±3.3 µM	59±4.8 µM

IC_50_ values were determined from the dose response data (Fig. 5) using a nonlinear least squares analysis (GraphPad Prism, v. 5.0). (*), p<0.05 for IC_50_ values for ATB-BMPA binding to GLUT4 vs. GLUT1 as determined by the Student's t test.

### Kinetic analysis of PI effects on ATB-BMPA labeling of LDM

**Figure 6:**
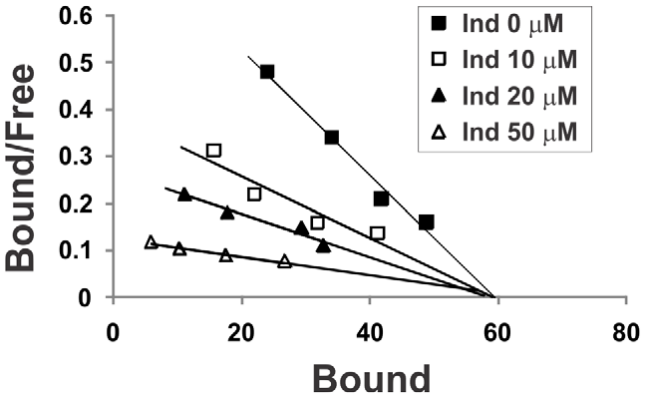
Indinavir competitively inhibits ATB-BMPA binding to GLUT4 in isolated low-density microsomes. The indicated concentrations of indinavir (Ind) were added to 50 µg of LDM for 10 min at room temperature. ATB-BMPA (50, 100, 200, 300 µM final concentration) was then added for an additional 10 min. Samples were UV irradiated and processed as described in “Materials and Methods”. The data are expressed as Scatchard plots. Bound and Free represent the ATB-BMPA bound to GLUT4 and ATB-BMPA free in solution, respectively.

Due to the presence of two distinct glucose binding sites in GLUTs on opposite sides of the cell membrane, the binding of PIs to the cytoplasmic glucose binding domain will appear non-competitive when zero trans 2DG uptake is measured since the tracer glucose analog does not have access to this site prior to facilitative transport. With demonstration that PIs interfere with the ATB-BMPA labeling of the cytoplasmic glucose binding site, we next determined the kinetic behavior of this effect. As shown in Figure 6, indinavir competitively blocks ATB-BMPA binding to GLUT4 in isolated LDM. The B_max_ (horizontal intercept) was constant while the K_d_ values (-1/slope) progressively increased with indinavir concentration. The calculated K_d_ values were 75, 152, 218, and 510 µM for 0, 10, 20, and 50 µM indinavir, respectively. The K_I_ of indinavir, calculated as described in “Materials and Methods” was 8.2 µM. This is somewhat lower than the K_I_ of 15 µM that was previously calculated from inhibition of zero trans glucose uptake in primary rat adipocytes [28]. The apparent difference between these values may reflect the influence of PI permeability and/or transport across the membrane.

## Discussion

Efforts to understand the mechanisms for altered glucose homeostasis in HIV infected patients have been limited by the complexity of interacting environmental, genetic, treatment and disease-related factors involved. Nevertheless, it is well established that antiretroviral therapy directly contributes to the development of diabetes [4] . Among the various antiretroviral agents in clinical use, HIV protease inhibitors are known to influence peripheral glucose disposal, hepatic glucose production, and insulin secretion [29]. Contrary to the initial speculation that induction of insulin resistance is a shared feature of all PIs, subsequent investigation has shown that individual agents within this drug class have differing effects on glucose homeostasis, both *in vitro* and in treated patients. The initial identification and characterization of GLUT4 as a direct molecular target of PIs was performed using indinavir [25]. The isoform selectivity of this drug (i.e. the ability to block GLUT4 activity with no effect on GLUT1) was established in *Xenopus* oocytes heterologously expressing either of these glucose transporters [28]. While it has been generally assumed that all PIs possess the same degree of isoform selectivity as indinavir, direct comparisons of glucose transport blockade in GLUT1 versus GLUT4 expressing cells have been lacking. The binding affinity (K_I_) of indinavir for GLUT4 in the oocyte system (50 µM) differs from that observed in primary adipocytes (15 µM). While the basis for this difference is unknown, contributing factors may include subtle structural differences in the expressed transporter due to lipid composition, assay temperature, the presence of additional proteins, or other factors. It was therefore necessary to directly compare the ability of both first generation and newer PIs to alter GLUT1 versus GLUT4 activity. These data provide a more comprehensive assessment of similarities and differences in the behavior of these PIs on facilitative glucose transport.

Several observations related to the ability of PIs examined in this study to compete for endofacial ATB BMPA binding have direct relevance to understanding the metabolic toxicities of these drugs in antiretroviral treatment regimens. Importantly, few studies to date have directly assessed the relationship between intracellular PI concentrations and impaired glucose uptake. Whether PI import occurs via simple diffusion or through mediated transport, sufficient drug levels may be present within the cytosol even when serum levels are low [30]. In addition, while it has been assumed that all PIs possess the same degree of GLUT isoform selectivity as indinavir, several PIs including ritonavir do not appear to distinguish among these transporters. Thus, the effects of some PIs on glucose homeostasis in tissues that do not express GLUT4 (such as hepatic glucose production in the liver and glucose-stimulated insulin secretion from β-cells) may still be mediated by changes in glucose transport. Comparison of the effects of various PIs in these tissues may provide further insight into the mechanistic basis for altered glucose homeostasis. More comprehensive assessment of the ability of individual PIs to block each of the other known GLUTs may provide insight into glucotoxicities. While atazanavir has a more favorable metabolic profile relative to first generation PIs, the current studies demonstrate that at drug levels above those typically achieved during clinical use, the potential for significantly altering glucose transport exists. The inability of tipranavir to alter either ATB BMPA binding or 2DG transport further supports the role of peptidomimetic structure in mediating binding to GLUTs.

Understanding of the molecular basis for the development of insulin resistance in HIV infected patients treated with PIs has already contributed to success in developing drugs within this class that do not directly alter glucose homeostasis. Nevertheless, many of these newer agents including tipranavir are associated with dyslipidemia [31] and may therefore indirectly contribute to impaired insulin signaling. Furthermore, with the potential for development of viral resistance over time, the need for continued drug development remains. Characterization of the molecular interactions between candidate drugs and GLUTs will assist ongoing efforts for rationale drug design, not only for antiviral efficacy, but also for metabolic toxicity.

Beyond further understanding of the mechanisms for PI-mediated insulin resistance, the ability to distinguish compounds that selectively interact with GLUT4 from those that bind to both GLUT1 and GLUT4 suggests that it may be possible to identify small molecule inhibitors of each of the other known GLUTs [32]. The availability of specific pharmacologic inhibitors of these transporters would provide a means to further characterize the functional role of these isoforms prior to the induction of potential compensatory changes in gene knockout models.

The development and use of an ATB-BMPA based assay for labeling of the cytoplasmic glucose binding site of GLUTs provides potential uses for this agent that extends its traditional use to quantify cell surface levels of GLUTs. This includes elucidation of the functional significance of cytosolic binding of known GLUT-interacting proteins and the discovery of additional protein-protein interactions. Such applications may aid efforts to identify means to improve diabetes treatment in the wider context of non-HIV associated insulin resistance.
